# Application of Bioactive Thermal Proteome Profiling to Decipher the Mechanism of Action of the Lipid Lowering 13^2^-Hydroxy-pheophytin Isolated from a Marine Cyanobacteria

**DOI:** 10.3390/md17060371

**Published:** 2019-06-21

**Authors:** Ana Carrasco del Amor, Sara Freitas, Ralph Urbatzka, Olatz Fresnedo, Susana Cristobal

**Affiliations:** 1 Department of Clinical and Experimental Medicine, Cell Biology, Faculty of Medicine, Linköping University, 581 85 Linköping, Sweden; ana.carrasco@liu.se; 2 CIIMAR, Interdisciplinary Centre of Marine and Environmental Research, 4450-208 Matosinhos, Portugal; freitas.srf.09@gmail.com (S.F.); rurbatzka@ciimar.up.pt (R.U.); 3 Department of Physiology, Faculty of Medicine and Nursing, University of the Basque Country UPV/EHU, 48940 Leioa, Spain; olatz.fresnedo@ehu.eus; 4 Department of Physiology, Ikerbasque, Faculty of Medicine and Nursing, University of the Basque Country UPV/EH, 48940 Leioa, Spain

**Keywords:** thermal proteome profiling, mechanisms of action, bioactive compound, label-free quantitative proteomics, marine biodiscovery

## Abstract

The acceleration of the process of understanding the pharmacological application of new marine bioactive compounds requires identifying the compound protein targets leading the molecular mechanisms in a living cell. The thermal proteome profiling (TPP) methodology does not fulfill the requirements for its application to any bioactive compound lacking chemical and functional characterization. Here, we present a modified method that we called bTPP for bioactive thermal proteome profiling that guarantees target specificity from a soluble subproteome. We showed that the precipitation of the microsomal fraction before the thermal shift assay is crucial to accurately calculate the melting points of the protein targets. As a probe of concept, the protein targets of 13^2^-hydroxy-pheophytin, a compound previously isolated from a marine cyanobacteria for its lipid reducing activity, were analyzed on the hepatic cell line HepG2. Our improved method identified 9 protein targets out of 2500 proteins, including 3 targets (isocitrate dehydrogenase, aldehyde dehydrogenase, phosphoserine aminotransferase) that could be related to obesity and diabetes, as they are involved in the regulation of insulin sensitivity and energy metabolism. This study demonstrated that the bTPP method can accelerate the field of biodiscovery, revealing protein targets involved in mechanisms of action (MOA) connected with future applications of bioactive compounds.

## 1. Introduction

The identification of protein targets from novel bioactive compounds is one of the biggest challenges of the field of biodiscovery. The function of those proteins would define the MOA of any bioactive compound, predicting the mode of action at the cellular level, as well as possible secondary or harmful effects. Phenotypic screening was the principal strategy for drug and bioactive compound discovery until the 80s. This methodology has attracted renewed interest in connection with biodiscovery programs for terrestrial natural sources [1]. As an alternative, targeted screening had offered enormous success in drug discovery, but requires a preliminary rational approach to MOA and an extensive screening of compound libraries against specific purified proteins used as targets [2]. Therefore, target selection should be restricted to proteins that can be expressed, purified, and adapted for interaction assays. Those limitations are introducing an intrinsic bias in the research. Moreover, considering that an average proteomic analysis from an homogenous cell could identify around 2000 proteins [3], and that over 700 proteins have been estimated to be targeted by current drugs [4], the traditional targeted screening approach faces difficulties to offer complete responses to drug–target opportunities.

The target engagement of a bioactive compound in cells and tissue depends on its local concentration, which is governed by parameters such as absorption, distribution, metabolism and excretion; and its affinity, which is also regulated by structural factors, including activation state of the protein target, co-factors, and post-translational modifications [5]. The challenge of evaluating those parameters in the cellular environment was solved by the development of the cellular thermal shift assay (CETSA) [6]. The biophysical principle of increase of thermal stability to unfolding of proteins in complexes compared to individual soluble proteins was the basis of CETSA [7]. The next step, extending the resolution power of this methodology to any possible protein targets within a cell, is offered by the TPP method [8]. The TPP method enables the analysis of the thermal stability of a proteome by applying quantitative mass spectrometry based on isobaric tandem mass tag 10-plex reagents. The method has been applied to study drug–target interaction [9,10], protein–substrate interaction in complex samples [11], and protein degradation [12].

The aim of biodiscovery is to understand the MOA of an array of newly discovered chemical compounds with possible bioactivity, limited structural characterization, and absence of any mechanistic knowledge. Identifying protein targets capable of interacting with the compound inside the cells is a huge challenge. In marine biodiscovery, cyanobacteria are recognized as being an interesting resource for obtaining novel compounds with applications in the field of human health. Cyanobacteria synthesize a wide variety of bioactive compounds with antimicrobial, antiviral and anticancer activity, among other things. Most secondary metabolites of cyanobacteria are lipopeptides, amino acids, fatty acids, macrolides, and amides. Although small compounds of cyanobacterial origin have been revealed to have activities of interest for application in pharmacology or as nutraceuticals, the strategies for elucidating MOA are still based on methods with low resolution [13].

In this study, we propose that implementation of the TPP methodology is applicable to novel bioactive compounds. The key implementation aims to gain in specificity and sensitivity for compound with limited chemical characterization. It should be considered that the TPP method was targeted to well-characterized drugs or druggable compounds. As phenotypic screening is the most common strategy for selecting positive candidates for bioactivity, chemical characterizations are not available at the early stages. The hydrophilic or hydrophobic nature of a new compound could compromise its interaction with biological membranes such as microsomes as a result of cellular lysis and fractionation. We evaluate the initial centrifugation step that determines the subproteome subjected to thermal shift analysis. Here, we present a method named bioactive thermal proteome profiling (bTPP). This is an improvement of the TPP method enabling sensitive analysis of protein targets across a proteome in any novel bioactive compound. To demonstrate the applicability of bTPP to the field of biodiscovery, we studied the protein targets of 13^2^-hydroxy-pheophytin a, a chlorophyll derivative with novel lipid-reducing activities that has recently been isolated from a marine cyanobacteria [14]. Given that this molecule is produced in high quantities in *Spirulina*, and is approved for human consumption, it is possible that a nutraceutical with anti-obesity activity may be developed in the future [14]. The identification of the direct targets of 13^2^-hydroxy-pheophytin a (hpa) in HepG2 liver cells and the discussion of possible MOA will provide important information in terms of its applicability.

## 2. Results

### 2.1. Implementation of the Novel Methodology, bTPP 

**Figure 1:**
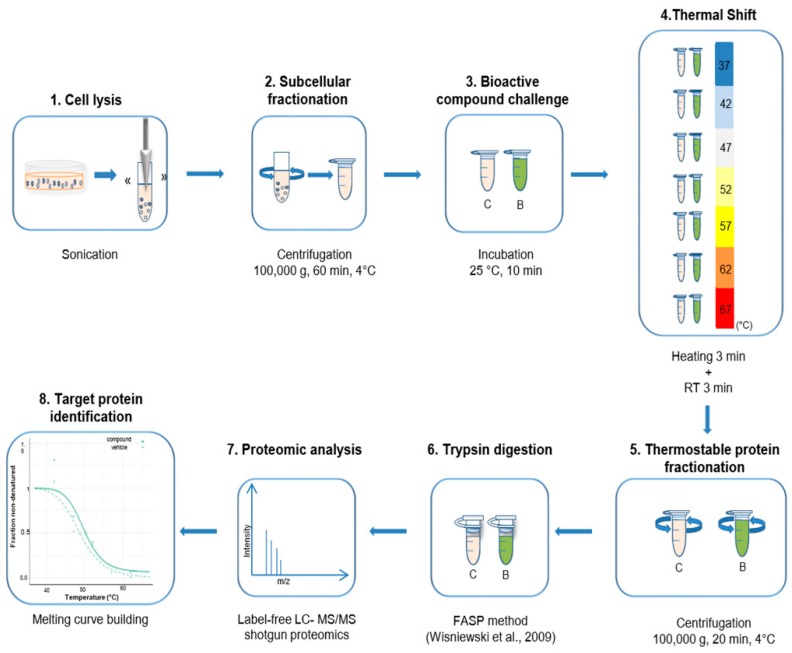
Outline of the bTPP workflow. (**1**) Cell lysis: performed using sonication with a vertical tip in the cell suspension. (**2**) Subcellular fractionation: soluble subproteome was collected in the supernatant after centrifugation at 100,000 *g*, for 60 min at 4 °C. (**3**) Bioactive compound challenge: the soluble protein was incubated with compound or vehicle at 25 °C for 10 min. (**4**) Thermal shift assay: performed at 7 temperatures between 37 °C and 67 °C for 3 min, and at RT for 3 min. (**5**) Thermostable protein fractionation: the studied sample was collected after centrifugation at 100,000 *g* for 20 min at 4 °C. (**6**) Trypsin digestion: the FASP method was applied. (**7**) Proteomic analysis: the peptides were separated by label-free LC-MS/MS and analysis by shotgun proteomic. (**8**) Target protein identification: protein data analysis was used to fit the melting curves of each protein.

We presented the bTPP method which is a TPP-based method able to identify the protein targets that interact with a bioactive compound. In particular, this method does not require preliminary knowledge of the chemical structure or function of a bioactive compound (Figure 1). 

**Figure 2:**
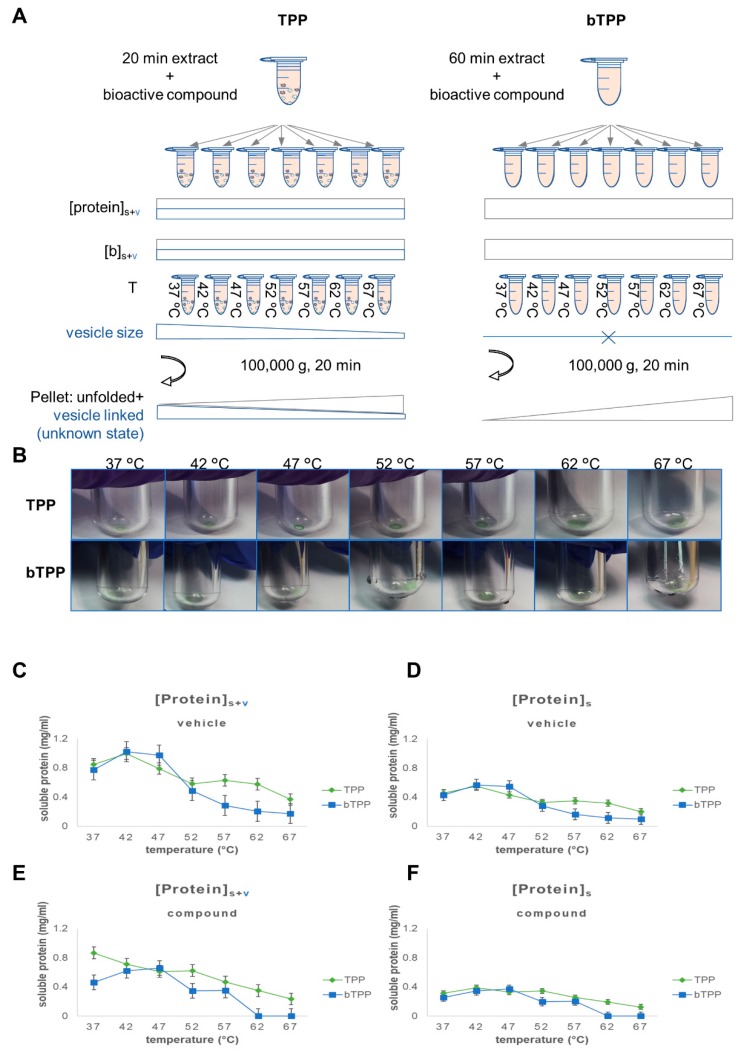
Characterization of the differences between the soluble subproteomes utilized in TPP and bTPP. (**A**) Schema summarizing the variation in the parameters: concentration of soluble protein, vesicular-associated protein, and bioactive compound in response to the thermal shift assay using both methodologies. (**B**) Pictures of the pellets after thermal shift assay centrifugation. (**C**,**E**) Representation of the total protein concentration from the supernatant of TPP and bTPP for each temperature after the second centrifugation step. (**D**,**F**) Representation of total protein concentration by % of soluble protein (obtained by Gene ontology (GO) classification) and divided by 100 for each temperature in TPP and bTPP.

We attempted to apply the TPP method as described for drug discovery [8]. We utilized ö, which has lipid-reducing activity and was extracted from marine cyanobacteria, as the test compound; it exhibited a green color in solution. The compound was dissolved in dimethyl sulfoxide (DMSO) due to its hydrophobicity. The first visual observation was the accumulation of hpa in the pellet following thermal shift assay centrifugation, which is defined in the TPP method as 100,000 *g* for 20 min (Figure 2). We hypothesized that, with the thermal shift incubation, the hydrophobic compound would be accumulated in the microsomes that were present in the soluble fraction. It has been described in the literature that the sedimentation of microsomes requires a centrifugation force at least equivalent to 100,000 *g* for 60 min [15,16,17]. Nevertheless, the TPP method applies only 100,000 *g* for 20 min to define the soluble fraction, and this fraction therefore contains insoluble microsomal vesicles [18]. The TPP method contains a second centrifugation step of 100,000 *g* for 20 min after the thermal shift assay, which would also precipitate additional microsomal vesicles. The TPP method is not able to discriminate, based on centrifugation, between an increase in protein instability caused by thermal effects and microsomal membranes precipitated with their sedimentation coefficient (Figure 2A). Thus, the first parameter to be modified is the definition of soluble subproteomes as a soluble protein free from microsomes obtained after centrifugation at 100,000 *g* for 60 min [15].

The second modification aimed to reduce the time, cost and processing efforts without compromising the robustness of the method. Our new temperature scale still covered the range from 37 °C to 67 °C, as with the original method, but we selected only 7 temperatures, including: 37 °C, 42 °C, 47 °C, 52 °C, 57 °C, 62 °C and 67 °C (Figure 1).

The final modification involved applying label-free quantitative mass spectrometry instead of multiplexed labelled quantitative mass spectrometry. This modification fits the purpose of the bTPP method, gaining the flexibility to be applied to several compounds in parallel and facilitating comparative analysis for more extended biodiscovery studies over extended periods of time.

### 2.2. Comparative Analysis of Protein Targets Applying TPP and bTPP

To validate our hypothesis, we compared the TPP and bTPP methods with a cell culture model of HepG2 cell homogenates and hpa as the bioactive compound. All of the thermal shift assays were performed at fixed concentrations of the protein and the bioactive compound. First, we deconvoluted the theoretical adjustment of the parameters protein, and bioactive compound, along with the thermal shift assay. This included changing the quality and quantity of the studied subproteome, as well as the availability of the bioactive compound (Figure 2A). 

In the TPP method, the concentration of the protein during the thermal shift incubation assay was distributed between the soluble fraction and the microsomal vesicles in the solution. The concentration ratio between both protein fractions is likely to remain constant at different temperatures. On the other hand, the concentration of the bioactive compound was distributed between soluble compound and compound embedded in the microsomes, as observed in the pellets following thermal shift assay centrifugation (Figure 2B). The concentration of the bioactive compound decreased to below the ideal concentration for the assay. Moreover, a temperature-dependent decrease was expected in the size of the vesicles. The concentration of protein in the vesicular fraction should be constant, but a higher number of smaller vesicles are expected at higher temperatures. Finally, the thermal shift assay centrifugation will cause the precipitation of unfolded soluble proteins along with the microsomes. Vesicular fractions would be precipitated depending on their specific sedimentation coefficient. An increase in temperature would reduce the vesicular size, and a higher sedimentation coefficient would be required to precipitate the smaller vesicles. Therefore, increase in temperature is associated with a decrease in the precipitation of the protein from the vesicular fraction (Figure 2A,B for TPP).

For the bTPP method, the schema shows that the concentration of protein or bioactive compound remains constant with the increase of temperature, a fact which constitutes the conceptual basis of any TPP-based method. In our method, only the protein is soluble, as the vesicular fraction has already been sedimented through the cellular fractionation. The proteins accumulated in the pellets after the thermal assay correspond to thermal unfolded proteins (Figure 2A,B for bTPP). 

The experimental data confirm this theoretical prediction. In the control samples, the decrease in protein concentration is temperature dependent in the bTPP for both total protein (soluble and vesicular fraction) and soluble protein, whereas a bimodal solubility pattern is observed for TPP (Figure 2C,D). In the presence of the compound, the protein thermostability limits are higher for TPP than for bTPP. This is most likely a consequence of the variation in the concentration of the bioactive compound when the TPP method is applied (Figure 2E). Both TPP and bTPP show a similar thermostability profile when exclusively comparing the soluble fraction that is available to interact with the compound (Figure 2F). 

**Figure 3:**
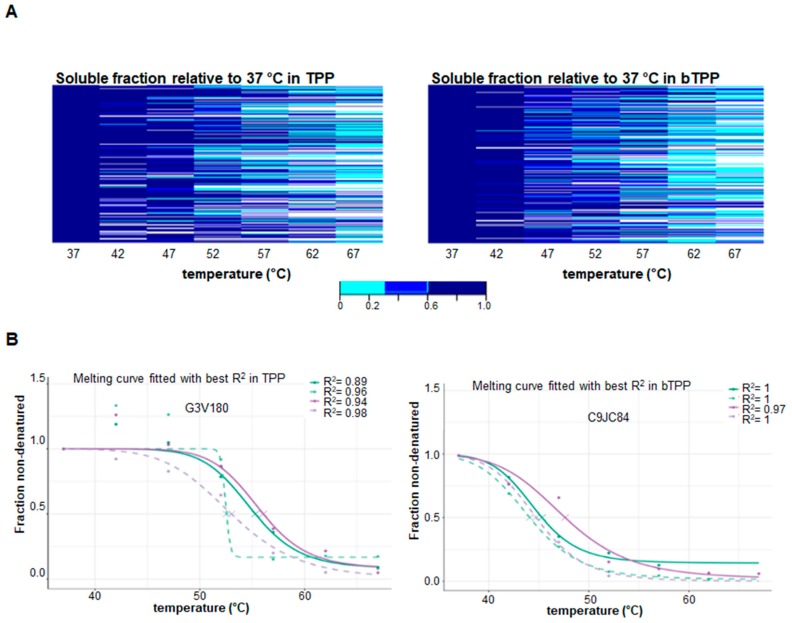
Thermal proteome profiling using TPP and bTPP. (**A**) Heat map of the protein thermostability in TPP, and bTPP. The colors show the range of protein abundance of the soluble fractions normalized to the soluble fraction at the lowest temperature. The soluble fraction here is composed of soluble protein after thermal shift assay and centrifugation. (**B**) Examples of melting curves fitted with the best R^2^ in both sets; the filter criterion was R^2^ > 0.8.

**Figure 4:**
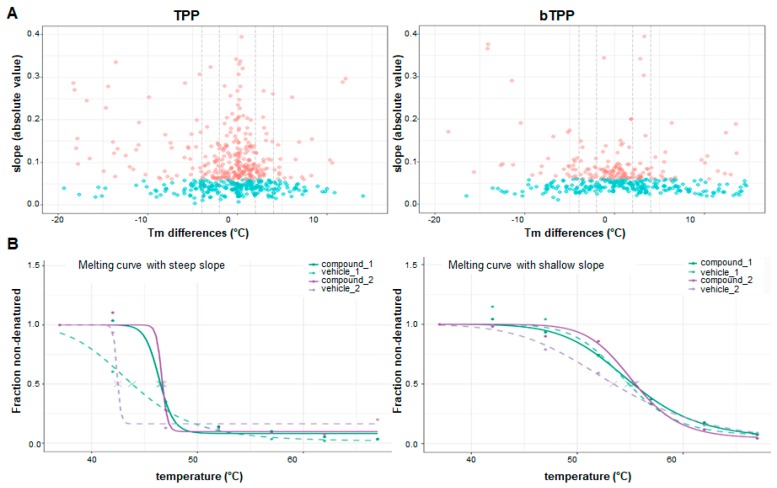
Differences in melting point between TPP and bTPP. (**A**) Volcano plots shown melting point differences between the two vehicle conditions versus the absolute slopes for the two vehicles and the two compound data sets. Proteins with an absolute slope below 0.06 are plotted in blue. (**B**) Example of a melting curve with a steep slope (left) and one with a shallow slope (right). Melting point reproducibility is dependent on the slope of the melting curve, with shallow slopes indicating less reproducibility.

The analysis of the soluble proteomes utilized in both methods showed different patterns in the heatmaps. For the TPP method, the map indicates a higher precipitation of membrane-associated proteins at lower temperatures. For the bTPP method, there was a greater abundance of soluble proteins at lower temperatures (Figure 3A). Both TPP and bTPP followed a sigmoidal trend, making it possible to calculate the melting curves that were fitted with the best R^2^ (Figure 3B). Target identification is based on the shift in melting temperature (Tm) induced by the ligand, and is dependent on the steep slope of the curve. At least 77% of all proteins showed curves with steep slopes in both methodologies (Figure 4A). Examples of melting curves with steep and shallow slopes are displayed in Figure 4B. 

The number of identified and quantified proteins were similar in both methods, at approximately 2500 proteins. The protein identification confirms that both datasets were associated with different subproteomes. The number of soluble proteins was lower in the subproteome that was utilized for the TPP method than in that used for the bTPP method. The sets of target proteins obtained using both methods differed in number and type of targets, with 19 proteins by TPP and 9 proteins by bTPP (Table 1 and Table 2).

### 2.3. Deciphering the MOAs for 13^2^-Hydroxypheophytine a by bTPP

**Figure 5:**
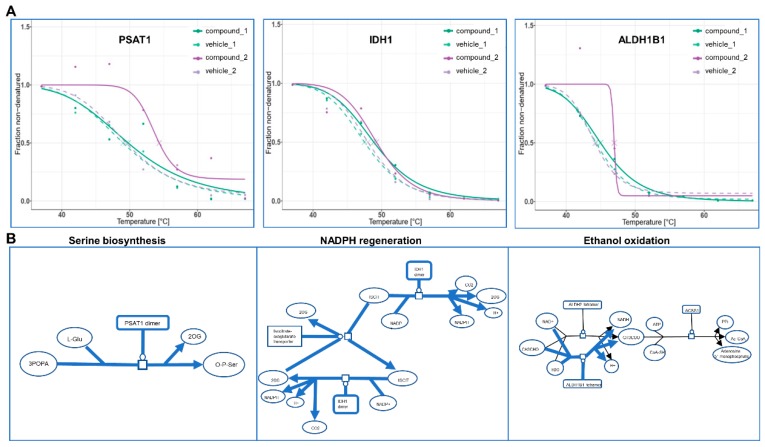
Target proteins and mechanism of actions based on bTPP analysis. (**A**) Melting curves of 3 of the target proteins. (**B**) Pathways of phosphoserine aminotransferase (left), isocitrate dehydrogenase (middle) and aldehyde dehydrogenase X (right).

The bTPP method was our method of choice for revealing the protein targets in our test compound, 13^2^-hydroxypheophytine a. Although the compound was characterized in parallel with its analysis by bTPP, neither chemical nor functional characterization are presumed or required prior to bTPP analysis. The melting curves of the proteins that met all of the quality criteria for both biological replicates were defined as the target proteins. From approximately 2500 proteins analyzed, only 9 target proteins were determined (Figure 5A). These proteins include: 40S ribosomal protein SA (RPSA), which is involved in a wide variety of biological processes including cell adhesion, differentiation, migration, signaling, neurite outgrowth and metastasis; fibrinogen gamma chain (FGG), which is a signaling binding receptor; isocitrate dehydrogenase (IDH1), which is a peroxisomal matrix protein, the enzyme of which catalyzes the reversible oxidative decarboxylation of isocitrate to yield α-ketoglutarate; heat shock protein beta-1 (HSPB1), which is a molecular chaperone and plays a role in stress resistance and actin organization; 26S proteasome regulatory subunit 6A (PSMC3), which is part of the ATP-dependent degradation of ubiquitinated proteins; aldehyde dehydrogenase X (ALDH1B1), which is the enzyme participating in the metabolism of corticosteroids, biogenic amines, neurotransmitters, and lipid peroxidation; the cell division control protein 42 homolog (CDC42), which participates in the regulation of the cell cycle; tubulin alpha-1B chain (TUBA1B), which is a structural protein in the cell; and phosphoserine aminotransferase (PSAT1), which is involved in amino acid synthesis. The targets resulting from the bTPP analysis were investigated for any implications on molecular pathways and cellular functions that might offer initial clues to deciphering the MOA of this compound after interaction with liver cells. The most relevant pathways discussed include serine oxidation, NADPH regeneration, and ethanol oxidation (Figure 5B).

## 3. Discussion

We present a TPP-based method that could provide an unbiased identification of target proteins in bioactive compounds without any preliminary information about chemical, functional or phenotypical characterization. The development of the method was specifically oriented towards novel compounds found in the course of biodiscovery. Here, we present a proof-of-concept by applying the novel method to a compound that has recently been isolated from a marine cyanobacteria due to its lipid-reducing activities [14]. The compound is a chlorophyll derivative, 13^2^-hydroxy-pheophytine a, which is present in marine and terrestrial organisms. The high rate of production of this molecule in *Spirulina* may enable the development of a future nutraceutical [14], and the identification of its protein targets will be an important step towards this aim.

The TTP is a thermal proteome profiling method, a high-throughput approach that makes it possible to examine an entire soluble proteome for its capability to interact with a drug in a single analytical experiment. Thermal shift-based methods have gained attention in the field of drug biodiscovery since the introduction of proteome analysis and protein target detection on the basis of mass spectrometry [8]. The improved method presented here, named bTPP, differs from the previous TPP methods developed for drug discovery in terms of the definition of the soluble fraction. This fraction is the subproteome analyzed by the thermal shift assay and is a pillar of the method. The robustness of the method and the reproducibility of its results would greatly depend on subjecting a single well-defined soluble proteome and compound to a series of incubations at range of increasing temperatures. If those parameters are modified by the methodological constraints, and the concentrations of the soluble proteins and the compound are variable, the proteomic analyses obtained by the thermal shift assays will not be able to be compiled in order to obtain target identification. 

By reviewing theoretical concepts in the field of cellular fractionation in connection with our findings, we determined that the sedimentation force applied to differentiate the soluble proteins from the vesicle-associated proteins using the TPP method [18] did not reach the minimal sedimentation force required to remove microsomal vesicles- by centrifugation [15,17]. The definition of the protein composition of a soluble fraction is not a universal concept. Rather, it is dependent on factors intrinsic to the sample: cell type, composition of the extraction solution, and the method applied for cellular homogenization. No less important are the extrinsic factors, including differential centrifugation, which aim to differentiate soluble from membrane-associated proteins. The soluble fraction is frequently obtained after applying 20,000 *g* for 20 min [8], or 100,000 *g* for 20 min of centrifugation [15,17,18]. These processes would barely be sufficient to clarify homogenized cells from the unbroken cells and the nuclear fraction. This type of soluble fraction still contains an abundant portion of organellar fractions such as mitochondria, lysosomes, peroxisomes and microsomal vesicles from the vesicular transport or plasma membrane. Proteins from these vesicle-rich fractions are semi-stable in solution, and would easily become unstable and have their precipitation prompted by the application of the additional steps of centrifugation force required to reach their specific centrifugation coefficients. This is the situation encountered by the TPP soluble fraction when a second centrifugation step at 100,000 *g* for 20 min is added in order to separate the unfolded proteins from the soluble proteins [18]. Therefore, in the TPP method, the classical microsomal fraction is part of the soluble fraction that is incubated with a bioactive compound. The presence of vesicle-associated proteins in the studied subproteome interferes with the expected results at different levels. 

First, the TPP method is based on the incubation of the soluble sample at a fixed concentration that is close to the IC_50_ of the compound at a series of increasing temperatures. The semi-stable soluble proteins after 20 min centrifugation contain vesicle-associated proteins. Those vesicles can potentially interact with hydrophobic compounds and entrap them within the membranes. This was the case for our test compound. The first consequence of this is that the concentration of the compound available for direct interaction with soluble proteins would shift away from the IC_50_, and the concentration would therefore be unknown. On the other hand, the compound inserted into the membranes could also interact with the soluble proteins, potentially leading to precipitation in association with the membrane.

Secondly, the fraction of the compound remaining in the solution would interact with the soluble proteins as predicted by TPP but would also interact with the membrane proteins in the vesicles that were not able to be evaluated using the method. Such interactions would further reduce the opportunities for interaction with the soluble proteins, which are the only proteins under evaluation. This is a second mechanism that modifies both the predicted concentration of the proteins during incubation and the predicted concentration of the compound.

Thirdly, the increasing temperature of successive incubations would affect the vesicles and their fluidity. It should at least be considered that the heterogeneity of the population of microsomal membranes would vary. At higher temperatures, there is expected to be an increase in the proportion of smaller vesicles. Smaller vesicles require a higher sedimentation force than bigger ones. Therefore, at higher temperatures, it is expected that there will be smaller vesicles in the solution and a reduction in the precipitation of the microsomal fraction compared to that observed at lower temperatures. This parameter is a variable that will affect the precipitation independently from the thermal shift factor or the total time of the centrifugation, and it will vary from temperature to temperature. In summary, applying the concept of the thermal shift to a soluble fraction obtained below the sedimentation coefficient for microsomal membranes will add many new variables to the system that are not considered by the method. Our results showed that this reduces the specificity and sensitivity required for an unbiased identification of the protein targets of our chosen test compound. Therefore, we developed the bTPP by modifying the criterion for protein solubility to require a centrifugation step of 100,000 *g* for 60 min, which is equivalent to the classical criterion for the precipitation of a microsomal fraction.

The bTPP method was our method of choice for revealing the protein targets of our test compound following confirmation that the parameters affecting the thermal shift analysis by bTPP were exclusively dependent on the interaction capability of the studied bioactive compound with the subproteome of the soluble proteins, and that the incubation at the different temperatures would not interfere with or cause variation in the concentration of the compound or the soluble protein. Here, we applied the bTPP method for our test compound without considering any preliminary information regarding its chemical structure, or evaluation or interpretation of the data from any functional assays. Nine proteins were assigned as its cellular targets in hepatocytes. In making a first attempt to discover any functional application of the compound, these targets were integrated in a map of functional pathways.

PSAT1 has already been described as a promising target for anticancer therapy [19]. This enzyme, which is involved in serine biosynthesis, has been associated with the metabolism of cancer, as extracellular serine may be sufficient to maintain cancer cell proliferation [20]. It has been proposed to be an oncogene with a significant role in cancer progression, inducing up-regulation of cyclin D1 via GSK3beta/beta-catenin pathway, leading to the acceleration of the cell cycle [21]. From a physiological perspective focusing on obesity and its related metabolic diseases, hepatic PSAT1 has revealed a novel function in the regulation of insulin sensitivity. The involvement of the nonessential amino acid serine in the regulation of insulin sensitivity opened lines of research into the targeting of PSAT1 for treatment of insulin resistance and type 2 diabetes in mice [22]. These effects on insulin-related disorders such as obesity and type 2 diabetes are also connected to two other protein targets, as revealed by bTPP with our test compound. For instance, ALDHs and their family of enzymes play a protective role in diseases related with obesity. ALDH2 has a role in the protection against diabetic cardiomyopathy, possibly via an Akt-GSK3b-mediated route, lending ALDH2 therapeutic promise in the management of diabetic complications [23]. Yu et al. [24] showed that the activation of the PKCε-ALDH2 regulatory axis may be a therapeutic target for treating obesity and type 2 diabetes in mice. Nonalcoholic fatty liver disease (NAFLD) is the most frequent chronic liver disease; alcohol dehydrogenase and aldehyde dehydrogenase collectively showed altered expression and function in the progression of nonalcoholic steatohepatitis (NASH) patients, which may also lead to significant alterations in the pharmacokinetics of substrate drugs. This information could be useful in making appropriate dosing adjustments for NAFLD patients taking drugs that are metabolized by these pathways [25]. 

Looking into IDHs, this target protein catalyzes the oxidative decarboxylation of isocitrate to α-ketoglutarate and reduces NAD(P)^+^ to NAD(P). IDH2 has been suggested as a potential therapeutic target in the treatment of type 2 diabetes and obesity due to its major role in modulating both insulin sensitivity and fuel metabolism in mice [26]. Moreover, Koh et al. [27] reported for the first time that cytosolic NADP^+^-dependent isocitrate dehydrogenase (IDPc) plays a critical role in fat and cholesterol biosynthesis, showing that transgenic mice with overexpressed IDPc exhibited fatty liver, hyperlipidemia, and obesity without an increase in caloric intake or change in diet composition, converting IDPc into a potential therapeutic target for abnormal fat synthesis. In summary, these 3 target proteins of 13^2^-hydroxy-pheophytine a are associated with beneficial properties towards obesity and obesity-related comorbidities. The next steps for progressing towards future application as a possible nutraceutical would be the carrying out of further research in order to validate the targets *in vivo* in a more complex organismal context. However, this study demonstrates that this methodology is able to accelerate the process between the biodiscovery of novel bioactive compounds to the revelation of protein targets involved in MOA of interest for intervention and application. 

## 4. Materials and Methods 

### 4.1. Reagents and Cell Culture

Reagents and medium were purchased from Sigma-Aldrich (Sant Louis, MO, USA), unless otherwise noted. PBS was purchased from Trevigen (Gaithersburg, MD, USA) and supplemented with 10 µL of ProteoGuard™ EDTA-Free Protease Inhibitor Cocktail (Takara Bio USA, Inc., Mountain View, CA, USA) per 1 mL. HepG2 cells were grown in EMEM medium supplemented with 8% fetal bovine serum (ATTC), 1675 mM L-glutamine, 85 U/mL penicillin, 85 μg/mL streptomycin of 80% confluence. Cells were harvested and centrifugated at 340 *g* for 2 min at 4 °C and resuspended in 50 mL PBS. After a second wash step, the cells were resuspended in 10 mL ice-cold PBS and centrifugated again at 340 *g* for 2 min at 4 °C. Washed pellets were either used directly or snap frozen in liquid nitrogen and stored at −80 °C until lysis. 

### 4.2. Selection of 13^2^-Hydroxypheophytine a as Test Compound

The compound selected for the proof-of-concept was 13^2^-hydroxy-pheophytin a (*hpa*), isolated from the marine cyanobacterial strain LEGE 07175 due to its lipid-reducing activity. The purity was estimated to ~99% by HR-ESI-MS and ^1^H-NMR analysis (Figure S1). The growth conditions of the cyanobacteria, as well as the chemical isolation methodology, are detailed in Freitas et al., 2019 [14]. 

### 4.3. Thermal Proteome Profiling Experiments in Cellular Protein Extracts

The experiments following the TPP method were performed as described in Franken et al. [18] with some modifications. Briefly, cells were resuspended in ice-cold PBS. The cells were homogenized in a Labsonic P disintegrator (B. Braun Biotech International, Göttingen, Germany) with an ultrasonic probe of 3 mm at 25% intensity and 0.5 cycles, with manual switches of 10 s on/5 s off, maintaining the sample in an ice bath. The sample was centrifugated at 100,000 *g* for 20 min at 4 °C [12]. The supernatant from this ultracentrifugation rendered the soluble subproteome that was applied in the TPP method. For the bTPP method, the homogenized sample was centrifugated at 100,000 *g* for 60 min at 4 °C. Protein concentration was determined by Bradford assay (Thermo Fisher Scientific, Waltham, MA, USA) [28]. From this point on, the process in both methods is identical. Two sets of thermal shift assays were performed using each methodology. The samples were incubated for 10 min at 25 °C. For the studied compound, incubation was performed at the compound IC_50_, and for the control, in the presence of the compound vehicle (DMSO). Seven aliquots of 100 μg of protein were individually heated for 3 min at different temperatures: 37 °C, 42 °C, 47 °C, 52 °C, 57 °C, 62 °C and 67 °C, followed by 3 min at room temperature. Subsequently, the samples were centrifugated at 100,000 *g* for 20 min at 4 °C. The supernatants were analyzed by label-free liquid chromatography-tandem mass spectrometry (nLC-MS/MS) (Thermo Fisher Scientific, Waltham, MA, USA). In accordance with the TPP method, two biological replicates for each set of the thermal shift assay were performed [18]. 

### 4.4. Filter Aided Sample Preparation (FASP) 

Protein samples were prepared according to Wiśniewski et al. (2012) [29]. The sample was diluted with 200 μL of 8 M urea in 0.1M Tris/HCl, pH 8.5 (UA) in 30 kDa microcon centrifugal filter unit (Merck Millipore, # MRCF0R030, Burlington, MA, USA). The centrifugal filters were centrifugated at 14,000× *g* at 20 °C for 15 min. The concentrates were diluted with 200 µL of UA and centrifugated at 14,000× *g* at 20 °C for 15 min. After discharging the flow-through, 100 µL of 0.05 M iodoacetamide was added to the column, mixed for 1 min at 600 rpm on a thermo-mixer (Eppendorf thermo mixer comfort, Hamburg, Germany), and incubated static for 20 min in dark. The solution was drained by spinning the columns at 14,000 *g* for 10 min. The columns were washed three times with 100 µL buffer UA and centrifugated at 14,000 *g* for 15 min. The columns were washed three times with 100 µL of 50 mM ammonium bicarbonate. Endopeptidase trypsin (Trypsin sequencing grade, Roche # 03708985001, Sigma-Aldrich, Sant Louis, MO, USA) solution in the ratio 1:100 was prepared with 50 mM ammonium bicarbonate (40 µL), dispensed and mixed at 600 rpm in the thermomixer for 1 min. These units were then incubated in a wet chamber at 37 °C for about 18 h to achieve effective trypsination. After 18 h of incubation, the filter units were transferred into new collection tubes. To recover the digested peptides, the tubes were centrifugated at 14,000 *g* for 10 min. Peptide recovery was completed by rinsing the filters with 50 µL of 0.5 M NaCl and collected by centrifugation. The samples were acidified with 10% formic acid (56302 Fluka, Sigma-Aldrich, Sant Louis, MO, USA) to achieve a pH between 3 and 2. Desalting was done using reverse-phase C18 top tips (TT2C18.96, Glygen, Columbia, MD, USA) using acetonitrile (ACN) (60% *v*/*v*) with formic acid (FA) (0.1% *v*/*v*) for elution, and vacuum dried (Savant SPD 1010, Thermo Fisher Scientific, Waltham, MA, USA) to be stored at −80 °C until further analysis.

### 4.5. Nano LC-MS/MS Analysis

The desalted peptides were reconstituted with 0.1% formic acid in ultra-pure milli-Q water, and the concentration was measured using a Nanodrop (ND 2000, Thermo Fisher Scientific, Waltham, MA, USA). The peptides were analyzed using a reverse phase nano-LC (liquid chromatography, Thermo Fisher Scientific, Waltham, MA, USA) coupled to a hybrid LTQ Orbitrap Velos Pro mass spectrometer (Thermo Fisher Scientific, Waltham, MA, USA). Each of the samples was separated using an Agilent 1200 Easy nLC (Agilent Technologies, Santa Clara, CA, USA) system with a nano-electrospray ion source (Thermo Fisher Scientific, Waltham, MA, USA). The peptides were trapped on a pre-column (NS-MP-10-C18-Biosphere, 5 µm particle size, 120 Å, 100 µm × 20 cm) and separated on an analytical column (NS-AC-10-C18-Biosphere, 5 µm particle size, 120 Å, 75 µm × 10.2 cm). A linear gradient of 2 to 40% buffer B (0.1% formic acid in acetonitrile) against buffer A (0.1% formic acid in water) was carried out with a constant flow rate of 300 nL/min, for a 90 min gradient. Full scan MS spectra were acquired in the positive mode electrospray ionization with an ion spray voltage of 2.4 KV, an RF lens voltage of 69, and a capillary temperature of 235 °C. This was acquired over an *m*/*z* of 380–2000 Da at a resolution of 30,000, and the 20 most intense ions were selected for MS/MS under an isolation width of 1 *m*/*z* units. Collision energy of 35 was used to fragment the ions in the collision-induced dissociation mode. 

### 4.6. Peptide and Protein Identification and Quantification

Proteome Discoverer (v2.1, Thermo Fisher Scientific, Waltham, MA, USA) was used for protein identification and quantification. The MS/MS spectra (.raw files) were searched by Sequest HT against the human database from Uniprot (73,928 entries). A maximum of 2 tryptic cleavages were allowed, the precursor and fragment mass tolerance were 10 ppm and 0.6 Da, respectively. Peptides with a false discovery rate (FDR) of less than 0.01 and validation based on q-value were used as identified. The minimum peptide length considered was 6, and the false discovery rate (FDR) was set to 0.1. Proteins were quantified using the average of the top three peptide MS1-areas, yielding raw protein abundances. Common contaminants like human keratin and bovine trypsin were also included in the database during the searches in order to minimize false identifications. The mass spectrometry proteomics data have been deposited in the ProteomeXchange Consortium via the PRIDE [30] partner repository with the dataset identifier PXD013227.

### 4.7. Analysis of TPP Experiments

Melting curves were calculated using a sigmoidal fitting approach with the R package TPP, as described in Franken et al. [18], with modifications. The fold changes were changed to correspond to the 7 temperatures, and the filter criteria for normalization were adjusted to this number of temperatures. 

The melting curves were fitted after normalization following the equation described in Savitski et al. [8], computed in R:

$$f\left(T\right)=\frac{1-plateau}{1+e^{-\left(\frac{a}{T}-b\right)}}+plateau$$

where *T* is the temperature, and *a*, *b* and “plateau” are constants. The value of *f*(*T*) at the lowest temperature *T*_min_ was fixed at 1. The melting point of a protein is defined as the temperature *T*_m_ at which half of the amount of the protein has been denatured. The quality criteria for filtering the sigmoidal melting curves were: (i) fitted curves for both vehicle- and compound-treated conditions had an *R*^2^ of >0.8; (ii) the vehicle curve had a plateau of <0.3; (iii) the melting point differences under both the control and the treatment conditions were greater than the melting point difference between the two controls; and (iv) in each biological replicate, the steepest slope of the protein melting curve in the paired set of vehicle- and compound-treated conditions was below −0.06. The NPARC of the R package was used to detect significant changes in the temperature-dependent melting behavior of each protein due to changes in experimental conditions [18]. The significance threshold was set at *p* < 0.05.

### 4.8. Pathway Analysis and Visualization

Pathway analysis was performed using Reactome Pathway analysis [31].

## 5. Conclusions

We have demonstrated that the thermal shift assay cannot be applied to a subproteome that contains soluble and vesicular fractions. We have deconvoluted the effects of a vesicular fraction in the protein sample, altering the concentration of protein and compound during the assay. These variations introduce uncertainties that challenge the principles of this methodology. Therefore, we presented an improved TPP method named bTPP based on a different postulate for protein solubility. The improvements guarantee that the concentration of proteins and compounds available for a raw thermal shift assay remains constant at any temperature. Finally, in a proof-of-concept experiment, we identified the protein targets in liver cells of 13^2^-hydroxy-pheophytine a, a compound recently isolated from a marine cyanobacteria due to its lipid-reducing activities. Three of these proteins have known regulations of insulin sensitivity and energy metabolism. 

## Figures and Tables

**Table 1:** Protein targets based on the bTPP method. Melting temperatures (Tm) and *p*-values calculated based on non-parametric analysis of response curves (NPARC).

Accession Name	Protein Name	Tm Control 1 (°C)	Tm Treatment 1 (°C)	Tm Control 2 (°C)	Tm Treatment 2 (°C)	*p*-Value
P08865 *	40S Ribosomal protein SA	45.74	47.16	45.32	49.00	0.0385
C9JC84	Fibrinogen gamma chain	44.09	45.32	44.74	47.63	0.1800
O75874	Isocitrate dehydrogenase [NADP] cytoplasmic	47.49	48.86	48.00	49.23	0.6307
P04792 *	Heat shock protein beta-1	45.23	49.82	47.94	51.40	0.0099
P17980	26S proteasome regulatory subunit 6A	44.67	47.01	44.18	46.65	0.1239
P30837	Aldehyde dehydrogenase X, mitochondrial	44.34	44.97	44.19	46.96	0.6788
P60953	Cell division control protein 42 homolog	46.93	47.70	47.09	51.92	0.6179
P68363 *	Tubulin alpha-1B chain	42.59	45.12	42.85	47.43	0.0242
Q9Y617	Phosphoserine aminotransferase	49.03	49.78	49.49	54.35	0.2253

* Proteins that passed quality criteria and *p*-values < 0.05.

**Table 2:** Protein targets based on TPP method. Melting temperatures (Tm) and *p*-values calculated based on NPARC.

Accession Name	Protein Name	Tm Control 1 (°C)	Tm Treatment 1 (°C)	Tm Control 2 (°C)	Tm Treatment 2 (°C)	*p*-Value
Q00341	Vigilin	47.04	50.62	46.91	47.60	0.2204
P42330 *	Aldo-keto reductase family 1-member C3	47.64	52.02	48.57	52.08	0.0001
G3V180	Dipeptidyl peptidase 3	52.55	55.30	53.01	56.06	0.6632
O14980	Exportin-1	47.84	49.29	49.28	51.48	0.1985
P00558	Phosphoglycerate kinase 1	53.07	53.91	53.03	54.06	0.9584
P07339 *	Cathepsin D	50.68	51.78	50.47	51.73	0.0045
P07814	Bifunctional glutamate/proline--tRNA ligase	43.83	46.51	42.48	46.70	0.2816
P08133	Annexin A6	52.45	53.03	52.99	55.30	0.9961
P09327	Villin-1	49.20	51.33	48.06	50.58	0.3429
P13674	Prolyl 4-hydroxylase subunit alpha-1	51.29	54.34	52.04	54.96	0.0954
P15559	NAD(P)H dehydrogenase [quinone] 1	49.21	50.48	49.08	49.30	0.8192
P30038	Delta-1-pyrroline-5-carboxylate dehydrogenase, mitochondrial	42.97	44.66	42.77	43.22	0.9284
P45954	Short/branched chain specific acyl-CoA dehydrogenase, mitochondrial	45.27	47.28	44.85	46.52	0.7475
P60709 *	Actin, cytoplasmic 1	44.10	48.42	41.84	45.14	0.0455
Q06210	Glutamine--fructose-6-phosphate aminotransferase [isomerizing] 1	47.31	48.12	47.14	47.55	0.9119
Q13347	Eukaryotic translation initiation factor 3 subunit I	46.89	48.21	46.64	46.91	0.6253
Q9NR45	Sialic acid synthase	53.17	53.41	53.22	54.67	0.4993
Q9Y490	Talin-1	49.93	51.85	51.75	53.92	0.1579
Q9Y696	Chloride intracellular channel protein 4	56.78	57.32	56.63	56.85	0.7306

* Proteins that met quality criteria with *p*-values < 0.05.

## Supplementary Materials

The following are available online at https://www.mdpi.com/1660-3397/17/6/371/s1, Figure S1: Spectrum of the 13^2^-hydroxy-pheophytine a, isolated from a marine cyanobacteria (LEGE07175) of the CIIMAR cyanobacterial culture collection (LEGE-CC), Figure S2: Melting curves from protein targets from bTPP.

## Abbreviations

TPP	thermal proteome profiling
MOA	mechanisms of action
CETSA	cellular thermal shift assay
bTPP	bioactive thermal proteome profiling
Tm	melting temperature
hpa	132-hydroxy-pheophytin a
DMSO	dimethyl sulfoxide
PBS	phosphate buffered saline
nLC-MS/MS	nano liquid chromatography-tandem mass spectrometry
FASP	filter aided sample preparation
RPSA	40S ribosomal protein SA
FGG	fibrinogen gamma chain
IDH1	isocitrate dehydrogenase
HSPB1	heat shock protein beta-1
PSMC3	26S proteasome regulatory subunit 6A
ALDH1B1	aldehyde dehydrogenase X
CDC42	cell division control protein 42 homolog
TUBA1B	tubulin alpha-1B chain
PSAT1	phosphoserine aminotransferase
NPARC	non-parametric analysis of response curves
