# Supplementary figures and images for: Application of Bioactive Thermal Proteome Profiling to Decipher the Mechanism of Action of the Lipid Lowering 132-Hydroxy-pheophytin Isolated from a Marine Cyanobacteria
Source: Mar Drugs. 2019 Jun 21;17(6):371. doi: 10.3390/md17060371 (PMC6627572; doi:10.3390/md17060371)

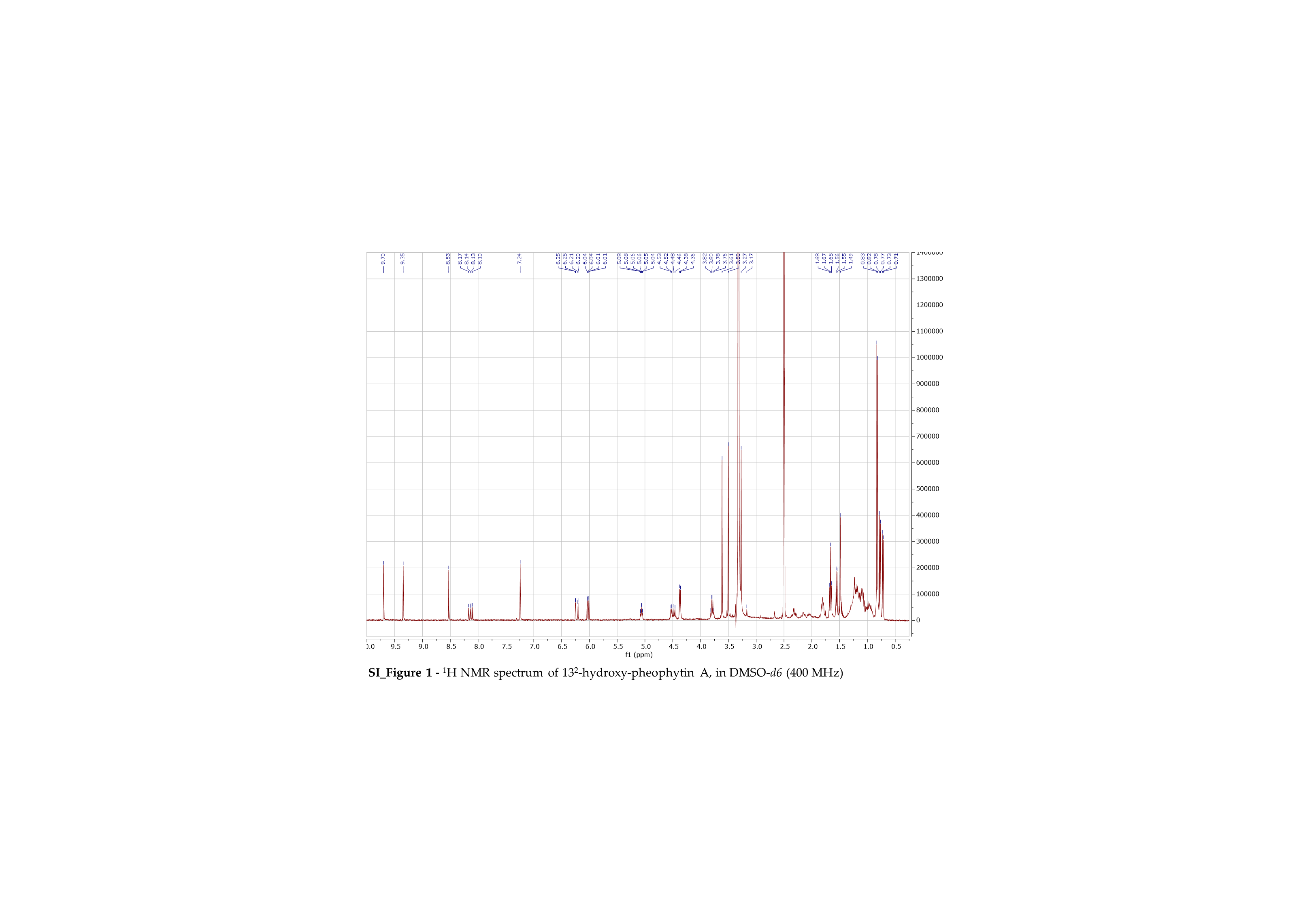

Supplement: Supplementary file 1 [file marinedrugs-17-00371-s001.zip › supplementary Figures/Si_Figure 1_190618.tif]

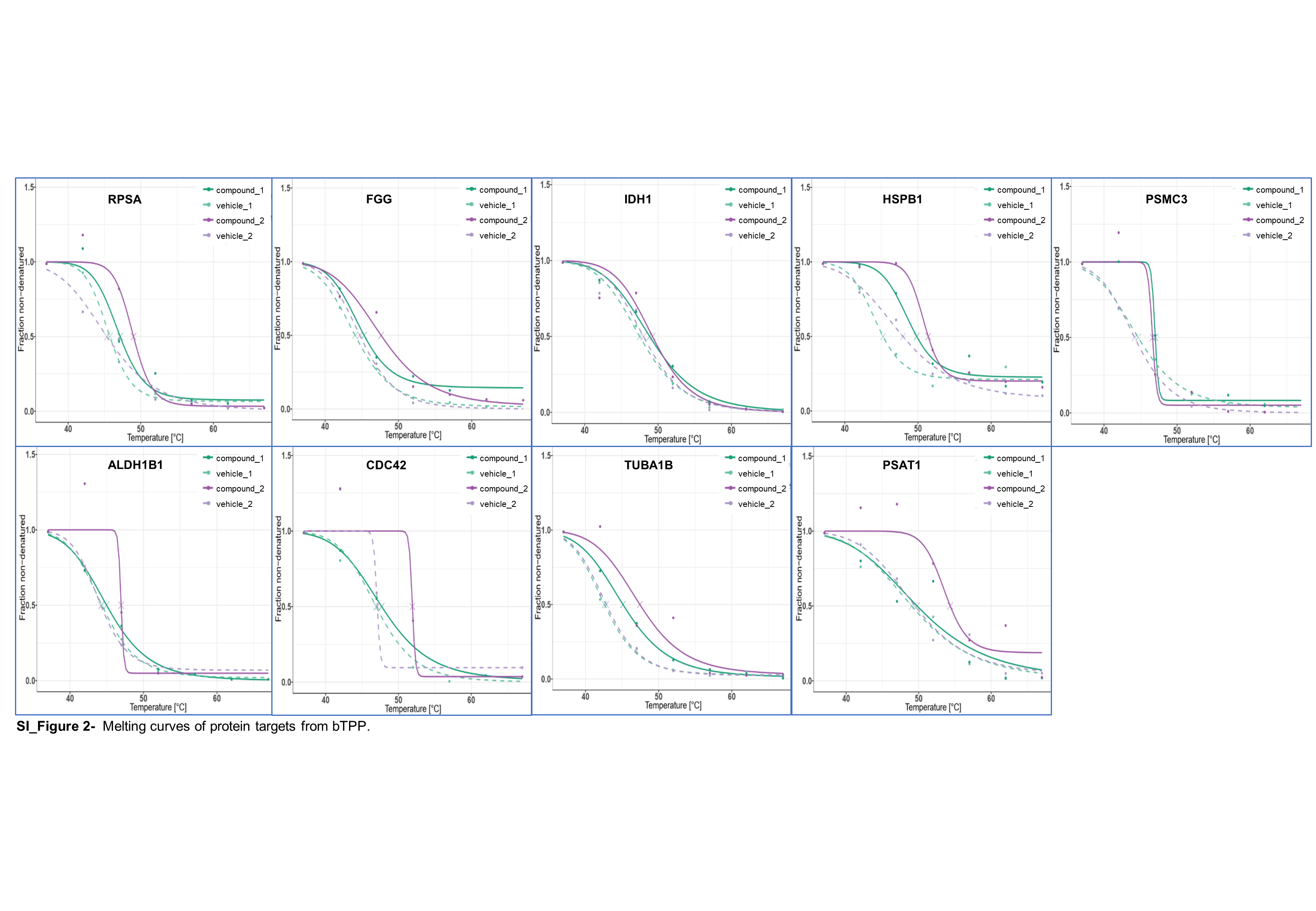

Supplement: Supplementary file 1 [file marinedrugs-17-00371-s001.zip › supplementary Figures/Si_Figure 2_190618.tif]
